# Absence of Tumor-Infiltrating Lymphocyte Is a Reproducible Predictive Factor for Sentinel Lymph Node Metastasis: A Multicenter Database Study by the Brazilian Melanoma Group

**DOI:** 10.1371/journal.pone.0148160

**Published:** 2016-02-09

**Authors:** João Pedreira Duprat, Eduard René Brechtbülh, Bianca Costa de Sá, Mauro Enokihara, Jose Humberto Fregnani, Gilles Landman, Marcus Maia, Felice Riccardi, Francisco Alberto Belfort, Alberto Wainstein, Luciana F. Moredo, Higino Steck, Miguel Brandão, Marcelo Moreno, Eduardo Miranda, Ivan Dunshee de Oliveira Santos

**Affiliations:** 1 AC Camargo Cancer Center, São Paulo, Brazil; 2 Medical School, São Paulo Federal University, São Paulo, Brazil; 3 Barretos Cancer Hospital, Barretos, Brazil; 4 Santa Casa de Misericórdia, São Paulo, Brazil; 5 Santa Rita Hospital, Porto Alegre, Brazil; 6 Sirio Libanês Hospital, Oswaldo Cruz Hospital, São José Hospital, São Paulo, Brazil; 7 Minas Gerais School of Medicine, Belo Horizonte, Brazil; 8 Mario Gatti Hospital, Campinas, Brazil; 9 AMO Clinic, Salvador, Brazil; 10 Medical School, Community University of Chapecó Region, Chapecó, Brazil; 11 Oncology Department, Pernambuco University, Recife, Brazil; University of Alabama at Birmingham, UNITED STATES

## Abstract

**Aims:**

The aim of this study is to confirm the function of tumor-infiltrating lymphocytes (TILs) in sentinel lymph node (SLN) metastasis.

**Materials and Methods:**

This retrospective study included 633 patients with invasive melanoma who underwent sentinel lymph node biopsy in 7 referral centers certified by the Brazilian Melanoma Group. Independent risk factors of sentinel node metastasis (SNL) were identified by multiple logistic regression.

**Results:**

SLN metastasis was detected in 101 of 633 cases (16.1%) and in 93 of 428 patients (21.7%) when melanomas ≤ 1mm were excluded. By multiple logistic regression, the absence of TILs was as an independent risk factor of SLN metastasis (OR = 1.8; 95%CI: 1.1–3.0), in addition to Breslow index (greater than 2.00 mm), lymph vascular invasion, and presence of mitosis.

**Conclusion:**

SLNB can identify patients who might benefit from immunotherapy, and the determination of predictors of SLNB positivity can help select the proper population for this type of therapy. The absence of TILs is a reproducible parameter that can predict SLNB positivity in melanoma patients, since this study was made with several centers with different dermatopathologists.

## Introduction

The Brazilian Melanoma Group (www.gbm.org.br) was founded in 1996 as a multidisciplinary team, comprising oncologists, dermatologists, plastic surgeons, pathologists, nurses, and any professional who was seeking the best strategies for the management of melanoma patients. In November 2000, a multicenter database was initiated, and after 14 years, the complete data on 1008 invasive melanomas have been analyzed, including sentinel lymph node biopsy (SLNB) findings.

The criteria for SLNB remain unknown, especially in thin melanomas [1–6]. Developed and emerging countries must direct their economic resources to balance the costs and benefits of medical procedures—particularly with regard to the MSLT-1 results (Multicenter Selective Lymphadenectomy Trial), which showed no survival benefits but highlighted the importance of staging, quality of life by regional disease control, and indications for adjuvant therapy [7].

This database was analyzed to establish Brazilian guidelines for SLNB, because each country requires specific and tailored recommendations [6,8,9]. The racial diversity in Brazil, whose miscegenation is a significant characteristic, makes its population unique–including Caucasians, Africans, Asians, and indigenous Brazilians [10,11].

The importance of certain histologic variables, such as Breslow thickness, ulceration, and the presence of mitosis, has been demonstrated with regard to SLNB positivity [1,5,12–15]. In this study, we examined the presence of tumor-infiltrating lymphocytes (TILs) as a prognostic factor, which has not been studied extensively. Thomas and colleagues reported the value of TILs in primary lesions in a large cohort from Australia, Italy, Canada, and the US. They recently concluded that the grade of TILs influences melanoma-specific survival [16]. Azimi et al. [14] and Taylor et al. [17] recently published the value of this factor in SNLB in a large cohort from a single-institution database. Also, in metastatic disease, the value of TILs was demonstrated in adoptive T-cell therapy using autologous TILs; in this study, the reinfusion of TILs and infusion of IL2 constituted an effective therapy [18].

In this multicenter study, including dermatopathology analyses from several professionals, we sought to determine the risk factors of sentinel lymph node (SLN) metastasis, especially TILs, to confirm them to be a reproducible parameter.

## Materials and Methods

This retrospective study included 633 patients with invasive melanoma who underwent SLNB in 7 referral centers certified by the Brazilian Melanoma Group from 2000 to 2015. It belongs to a larger database of 1,343 melanoma patients with and without sentinel lymph node biopsy. The data from each patient were stored in a provisional database reviewed by an attending physician and sent back to the melanoma center for correction due to errors or incompleteness; corrected data were included in the definitive database. A statistician or physician was responsible for analyzing the data and responding to any questions raised by the authors. The database used for analyses identified patients only by numbers. The study EC29/15 has been approved by A.C. Camargo Cancer Center Ethics Committee on Research.

After a consensus meeting in Brazil, melanoma SLNB was indicated for patients with cutaneous melanoma with a Breslow depth ≥ 0.75 mm or < 0.75 mm if presenting with Clark level IV or V involvement, regression, and ulceration. All cases < 0.75 presented those conditions [19]. All participating melanoma centers followed the Brazilian guidelines for the histological evaluation of sentinel lymph nodes (SLNs) [20]. Prior to surgery, patients were evaluated by lymphoscintigraphy to locate SLNs. In most cases, blue dye and a hand-held gamma probe were used to identify sentinel nodes intraoperatively (n = 583, 93.0%). The remaining cases used either one technique or the other.

Sentinel lymph nodes were examined as follows: 2 mm cross-sections of the main axis from the entire lymph node were paraffin-embedded. Three levels of each paraffin block were obtained. Four-micron sections were stained with routine hematoxylin and eosin (H&E); three spare unstained sections were subjected to immunohistochemistry for S100 protein, HMB45, and, whenever possible, Melan-A if no micrometastases were noted in the H&E-stained sections [21].

The following variables were recorded: age, gender, race, location, histological type, Breslow thickness, Clark level, ulceration, regression, vertical or radial growth phase, mitosis, lymph vascular invasion, perineural invasion, tumor-infiltrating lymphocytes, peritumoral inflammatory infiltration, satellitosis, and sentinel node status. For the purpose of this investigation, intratumoral lymphocytic infiltration was compared present versus absent. Brisk and non-Brisk (mild lymphocytic infiltrate) were lumped together.

The study population was characterized using descriptive statistics. The risk for sentinel node metastasis was estimated using the odds ratio (OR) and 95% confidence interval calculated by logistic regression using SPSS version 21.0. Stepwise forward selection was used for the multiple model. The confidence interval for the proportions was calculated by means of MedCalc 13.0.

The medical records were analyzed per the Declaration of Helsinki and ethical standards. The patients’ identities were preserved, as were their data, which were assessed only by the investigators. Informed consent was not necessary as it was a retrospective study and only clinical data from medical records were analyzed.

## Results

In the database for patients with and without SLNB, the proportion of patients that were submitted to SLNB was shown in Table 1. SLN metastasis was identified in 102 cases, yielding a positivity rate of 16.1% (95%CI: 13.1%– 19.6%) for all cases and 21.7% (95% CI: 17.5%– 26.6%) when melanomas ≤ 1mm were excluded. Table 2 describes the risk of SLN metastasis by study variable. The risk of SLN metastasis was greater for nonwhite patients (OR = 2.6; 95% CI: 1.3–5.03), melanoma in the leg (OR = 4.2; 95% CI: 1.3–14.4), nodular type (OR = 1.9; 95% CI: 1.1–3.3), acrolentiginous type (OR = 3.3; 95% CI: 1.8–6.0), presence of ulceration (OR = 3.1; 95% CI: 2.0–4.8), absence of regression (OR = 2.2; 95% CI: 1.2–4.0), vertical growth phase (OR = 4.8; 95% CI: 1.5–15.6), presence of mitosis (OR = 11.5; 95% CI: 2.8–47.6), lymph vascular invasion (OR = 6.9; 95% CI: 2.8–17.2), perineural invasion (OR = 3.6; 95% CI: 1.6–7.9), absence of TILs (OR = 1.5; 95% CI: 1.0–2.5), and satellitosis (OR = 3.6; 95% CI: 1.5–9.2).

**Table 1:** Proportion and positivity rate of sentinel lymph node biopsy according to Breslow categories (n = 1,073).

Breslow	N	SLNB procedure	Positive SLNB
**< 0.75**	409	98 / 409 (24.0%)	4 / 98 (4.1%)
**0.75–1.00**	155	107 / 155 (69.0%)	5 / 107 (4.7%)
**1.01–2.00**	238	208 / 238 (87.4%)	27 / 208 (13.0%)
**2.01–4.00**	158	135 / 158 (85.4%)	32 / 135 (23.7%)
**> 4.00**	113	85 / 113 (75.2%)	34 / 85 (40.0%)
**Total**	1,073	633 / 1,073 (59.0%)	102 / 633 (16.1%)

SLNB: Sentinel lymph node biopsy.

**Table 2:** Risk for sentinel lymph node metastasis by study variable (n = 633).

Variable	Description	N	(%)	OR (95% CI)
**Race**	White	587	(92.9%)	1.0
** **	Nonwhite	45	(7.1%)	2.6 (1.3–5.0)
** **	Missing	1		
**Gender**	Female	357	(56.4%)	1.0
** **	Male	276	(43.6%)	1.0 (0.7–1.6)
**Location**	Head and Neck	44	(7.2%)	1.0
** **	Trunk	257	(42.3%)	2.2 (0.7–7.6)
** **	Arm	116	(19.1%)	2.3 (0.7–8.4)
** **	Leg	190	(31.3%)	4.2 (1.3–14.4)
** **	Missing	26		
**Histological Type**	Superficial spreading	426	(71.6%)	1.0
** **	Nodular	99	(16.6%)	1.9 (1.1–3.3)
** **	Acrolentiginous	64	(10.8%)	3.3 (1.8–6.0)
** **	Lentigo maligna	6	(1.0%)	NA
** **	Missing	38		
**Breslow depth**	<0.75 mm	98	(15.5%)	1.0
** **	0.75–1.00 mm	107	(16.9%)	1.2 (0.3–4.4)
** **	1.01–2.00 mm	208	(32.9%)	3.5 (1.2–10.3)
** **	2.01–4.00 mm	135	(21.3%)	7.3 (2.5–21.4)
** **	>4.00 mm	85	(13.4%)	15.7 (5.3–46.6)
**Clark**	II	88	(14.1%)	1.0
** **	III	285	(45.6%)	1.7 (0.7–4.1)
** **	IV	219	(35.0%)	4.0 (1.6–9.6)
** **	V	33	(5.3%)	11.4 (3.9–33.4)
** **	Missing	8		
**Ulceration**	Absent	465	(73.8%)	1.0
** **	Present	165	(26.2%)	3.1 (2.0–4.8)
** **	Missing	3		
**Regression**	Absent	475	(75.9%)	2.2 (1.2–4.0)
** **	Present	151	(24.1%)	1.0
** **	Missing	7		
**Growth phase**	Radial	70	(11.1%)	1.0
** **	Vertical	559	(88.9%)	4.8 (1.5–15.6)
** **	Missing	4		
**Mitosis**	Absent	98	(16.0%)	1.0
	Present	516	(84.0%)	11.5 (2.8–47.6)
	Missing	19		
**Lymph vascular invasion**	Absent	607	(96.8%)	1.0
** **	Present	20	(3.2%)	6.9 (2.8–17.2)
** **	Missing	6		
**Perineural invasion**	Absent	598	(95.5%)	1.0
** **	Present	28	(4.5%)	3.6 (1.6–7.9)
** **	Missing	7		
**Tumor infiltration lymphocytes**	Absent	401	(64.4%)	1.5 (1.0–2.5)
** **	Present	222	(35.6%)	1.0
** **	Missing	10		
**Peritumoral inflammatory infiltration**	Absent	208	(33.3%)	1.0
** **	Present	417	(66.7%)	1.4 (0.9–2.2)
** **	Missing	8		
**Satellitosis**	Absent	608	(96.8%)	1.0
** **	Present	20	(3.2%)	3.6 (1.5–9.2)
** **	Missing	5		

Melanoma thickness was associated with SLN metastasis as follows: 0.75–1.00mm (OR = 1.2; 95% CI: 0.3–4.4), 1.01–2.00mm (OR = 3.5; 95% CI: 1.2–10.3), 2.01–4.00mm (OR: 7.3; 95% CI: 2.5–21.4), and >4.00mm (OR: 15.7; 95% CI: 5.3–46.6). Clark levels IV (OR = 4.0; 95% CI: 1.6–9.6) and V (OR = 11.4, 95% CI: 3.9–33.4) also correlated with an increased risk of metastasis. No significant difference was observed in mean patient age according to SLN status between those with (52.2 years; 95% CI: 48.9–55.5) and without metastasis (51.4 years; 95% CI: 50.1–52.6).

By multiple logistic regression, the absence of TILs (OR = 1.8; 95% CI: 1.1–3.0) was an independent risk factor for SLN metastasis, in addition to Breslow index (greater than 2.00 mm), lymph vascular invasion and presence of mitosis (Table 3).

**Table 3:** Independent predictive factors of sentinel lymph node metastasis according to the multiple logistic regression analysis (n = 608^*^).

Variable	Category	N	OR	(95% CI)
**Breslow**	< 0.75 mm	95	1.0	Reference
** **	0.75–1.0 mm	100	0.8	(0.2–3.5)
** **	1.01–2.00 mm	202	2.7	(0.9–8.0)
** **	2.01–4.00 mm	130	5.3	(1.8–15.8)
** **	>4.00 mm	81	10.2	(3.3–31.3)
**Lymph vascular invasion**	Absent	588	1.0	Reference
** **	Present	20	3.5	(1.3–9.1)
**Tumor-infiltrating lymphocytes**	Absent	388	1.8	(1.1–3.0)
** **	Present	220	1.0	Reference
**Mitosis**	No	98	1.0	Reference
	Yes	510	5.7	(1.3–24.3)

OR: Odds ratio, CI: Confidence interval.

Number of events considered in the model: 101 (sentinel lymph node metastasis).

(*) 608 cases had complete information of the four variables included in the model.

## Discussion

The validity of SLNB has continued to be debated extensively [1–6,8,19–22]. For instance, there are no consensus criteria on the indications for thin and thick melanomas, even among physicians who advocate the use of SLNB [23]. In countries such as Brazil, that strive to provide the best medical care with limited resources, efforts must be directed towards optimizing the indications for SLNB. Even if resources are not a concern, unnecessary surgeries can have undesirable side effects, such as lymphedema and paresthesia [24,25].

Based on MSLT I, which did not provide a clear survival benefit of the SLNB procedure, the primary goals are to achieve regional control and identify the patients who should receive adjuvant therapy [7]. The current indications for SLNB per the Brazilian Melanoma Group are Breslow depth ≥0.75 mm or <0.75 mm with Clark level IV or V, regression, and ulceration [19]. An analysis of SNLB parameters is essential for reestablishing its indications. In study population, in cooperation with several dermatopathologists, we found the absence of TILs to be a reproducible parameter for SLNB indication. But we have also reviewed other established indications.

We found that the absence of TILs was a predictive factor of SLN metastasis, independent of Breslow index, lymph vascular, and mitosis status. The value of TILs in the prognosis of melanoma has been discussed. A survival benefit is observed for primary lesions with the presence of TILs [14,16]. In the lymph node, a brisk reaction appears to be a prognostic influence in lymph node metastasis, decreasing the occurrence of SLN metastasis [26,27]. Many of these studies are single institution series, but in the present paper there are a number of dermatopathologists, so we have to stress the importance of the reproducibility.

The value of TILs is evident in therapeutic procedures, such as adoptive T cell therapy, with which tumors can regress on re-injection of TILs, especially in association with IL2 [18]. However, there are many types of lymphocytes (T cells, natural killers, dendritic cells, macrophages, and others) that are responsible for TILs—some of which promote tumor growth, whereas others impede it [28]. Thus, the prognostic significance of TILs must be demonstrated, in conjunction with our findings (analyzed by many dermatopathologists), which will show the reproducibility of this factor.

Notably, the presence of brisk TILs has been linked to a lower probability of positive SLNB in univariate and multivariate analyses; others authors have recently shown a similar association [14,17,29]. They specifically investigated marked intratumoral infiltration and found that the absence of TILs increased the risk of SLN positivity by 25-fold compared with their presence.

The findings in the literature are controversial regarding tumor thickness as an indication for SLNB. Some studies advocate SLNB for patients with a thickness 1 mm [1–4] or 0.75 mm [5,19], whereas other studies suggest that SLNB should not be performed in cases with a depth of less than 0.75 mm but is indicated for cases with a thickness of 0.75–1 mm in which the mitotic index is greater than 0 and there are ulceration and lymph vascular invasion observed [1,6]. There is a consensus that for lesions with a Breslow thickness > 4mm, SLNB helps only in regional control [23].

In our cohort, SLNB was performed for 69% of tumors with a thickness between 0.75 and 1 mm, in most patients with intermediate thickness (1–4 mm) 87%, and in 75% of those with melanomas that were thicker than 4 mm. In the group with a thickness under 0.75 mm, surgery was performed due to adverse prognostic factors, such as regression, ulceration, and Clark level IV or V, regardless of whether they were substantiated. Even with these bad prognosis factors in this group under 0.75mm, the SLN metastasis rate was 4.1%.

Kunte et al. [12] suggested SLNB for lesions that were <1mm thick only if they were associated with a poor prognostic factor, such as ulceration, regression, and Clark level IV or V. These authors noted a 6% rate of SLN metastasis in cases with a depth of less than 0.75 mm and an 8% metastasis rate in cases with a thickness between 0.75 and 1 mm. In the authors view, data do not justify the recommendation of SLNB for melanomas with a thickness of less than 0.75mm, even if mitosis is observed. Ulceration and lymph vascular invasion are rare in such thin melanomas. Moreover, Clark level and regression do not appear to be risk factors for SLNB (see below).

In our series, melanomas with a thickness between 0.75 and 1 mm had a 4.7% rate of SLN positivity, indicating that there is little value in recommending SLNB in these cases, unless mitosis, ulceration, lymph vascular invasion, or satellitosis is present [12]. Nevertheless, SLN metastasis in thicknesses of 0.75–1 mm is debated. Han et al. (2012) recorded 8% SLN metastasis rate in this range of thicknesses versus 5% and 13% with mitosis or ulceration [5].

Over time, Clark level has lost significance and was downgraded in the last AJCC stage system update in 2009 [30]. In our study, 9 cases with a thickness of less than 1 mm had a positive SLNB, of whom only 1 was Clark level IV, and none was Clark level V. Thus, we believe that Clark level should not be considered in indicating SLNB in thin melanomas. This conclusion is supported by White et al. [31], who found no relationship between Clark level and sentinel status by multivariate analysis.

There is no consensus on whether regression has an adverse or favorable impact on melanoma survival [3,32–34]. The reproducibility of regression impact is usually poor, primarily because pathologists have failed to agree on the criteria for the diagnosis of regression [35,36]. Our regression criteria stated that there should be complete absence of full-thickness tumor cells (even the *in situ* component) in a tumor segment that has been replaced by fibrosis. In addition, fibrosis and inflammatory reactions in parts of the melanoma were not considered regression. In a review by Requena et al. [36], full-thickness regression was linked to a worse prognosis. In our series, by multivariate analysis, there was no impact of regression on SLN positivity, and there was only 1case of regression with positive nodes, compared with 7 cases without regression, in the thin melanoma group. Most centers no longer indicate SLNB for thin melanomas [3,5,13,37].

Ulceration is a strong prognostic factor, regardless of tumor thickness. Thin melanomas with ulceration are rare, and patients have a high probability of developing local, regional, and distant metastases [12,14,35]. In our series, ulceration was highly associated with SLN metastasis by univariate analysis but was not significant in the multivariate analysis. Based on larger series [1,5,13,14], we believe that patients with ulceration should undergo SLNB, regardless of tumor thickness.

Mitotic activity was not discussed in previous recommendations as a predictive factor of SNL metastasis. Nevertheless, because mitotic activity is now considered an important prognostic factor [14,30] that indicates a greater probability of node metastasis, we included it in our analysis. Although mitotic activity is a significant prognostic factor in tumors that are less than 0.75 mm thick, its presence alone is insufficient to justify its use as an indicator for SLNB for such depths [5]—the results regarding the correlation between mitotic index and SLNB metastasis in tumors that are greater than 1 mm are contradictory [13,15]. Moreover, SLNB should only be considered in tumors that are 0.75–1 mm thick if mitosis is present [1]. In this study, mitotic index was an important prognostic factor for SLN metastasis in the univariate and multivariate analysis, and no case with positive SLNs without mitosis was observed in thin melanomas.

To sum up, the absence of TILs is a reproducible parameter that can be used as a predictive factor for positive SLNB in melanoma patients.
